# Risk factors for manipulation under anesthesia after total knee arthroplasty and subsequent revision arthroplasty: a Finnish register-based study of 154,883 patients

**DOI:** 10.2340/17453674.2026.46072

**Published:** 2026-06-22

**Authors:** Julius SALA, Joonas SIROLA, Antti JAROMA, Heikki KRÖGER, Reijo SUND

**Affiliations:** 1 Kuopio Musculoskeletal Research Unit (KMRU), Institute of Clinical Medicine, University of Eastern Finland (UEF), Kuopio; 2 Department of Orthopaedics, Traumatology and Hand Surgery, Kuopio University Hospital, Kuopio; 3 Health and Social Service System Research Unit, Department of Welfare State Research, Finnish Institute for Health and Welfare (THL), Helsinki; 4 Clinical Research Centre, Kuopio University Hospital, Kuopio, Finland

## Abstract

**Background and purpose:**

Stiffness after total knee arthroplasty (TKA) is a common early complication and multiple risk factors are recognized. We aimed to investigate the risk factors for manipulation under anesthesia after primary TKA and for the subsequent revision TKA in patients requiring manipulation using national healthcare registers.

**Methods:**

We used the comprehensive register data of the PERFECT project that included data from the Finnish arthroplasty register (FAR) and the Care Register of Health Care (CRHC). We excluded patients under 40 years old. The Aalen–Johansen estimator and Cox proportional hazards regression model were used in the risk assessment.

**Results:**

154,883 patients had primary TKA in Finland in 1999–2020 , of which 3,861 patients required manipulation within 1 year of primary TKA. The 1-year cumulative incidence of manipulation was 2.5%. In the multivariable analysis, female sex (hazard ratio [HR] 1.53, CI 1.42–1.64), diabetes mellitus (HR 1.19, CI 1.08–1.31), coronary artery disease (HR 1.25, CI 1.12–1.39), and hypercholesterolemia (HR 1.16, CI 1.06–1.28) were associated with an increased risk of manipulation. Increasing age was associated with a decreased risk of manipulation (multivariable HR 0.94 per year, CI 0.94–0.94). Patients requiring manipulation within 1 year of primary TKA had a significantly increased risk of revision TKA (HR 2.26, CI 2.05–2.50). The 10-year cumulative risk of revision TKA after manipulation was 15% (CI 14–16).

**Conclusion:**

Manipulation was more likely to be performed for females, relatively younger patients, and patients with diabetes mellitus, coronary artery disease, or hypercholesterolemia. Patients who had manipulation within 1 year of primary TKA had an increased risk of revision with a 10-year cumulative risk of revision of 15%.

Generally, the range of motion after total knee arthroplasty (TKA) is satisfactory [1]. Postoperative stiffness requiring manipulation under anesthesia occurs in 1.5–5.3% of cases [2-9]. Stiffness is commonly caused by arthrofibrosis [10]. Various risk factors for stiffness and manipulation after TKA have been proposed including low preoperative range of motion [6,11], younger age [3-6,8,9,11], female sex [5,8,9,12], high body mass index (BMI) [8,12], history of previous knee surgeries [3,5], current smoking [3,4,8,9,11], and diabetes mellitus [11,13]. Additionally, malposition and/or oversizing of TKA components have been found to increase the risk of postoperative stiffness [14].

While a number of studies have been conducted regarding the risk factors for stiffness after TKA, the risk factors for revision TKA in patients requiring manipulation have been less studied. The most common reasons for revision TKA are infection, aseptic loosening, and periprosthetic fracture [15,16]. Previous studies found that patients undergoing manipulation after TKA had a higher risk of revision TKA than general TKA patients [5,9,17]. Furthermore, the indication for revision TKA was less often infection and more often mechanical in manipulation patients compared with general TKA patients [5,9,17].

It was recently emphasized that manipulation rates vary considerably and more knowledge about the factors that influence the decision to perform manipulation is required [18]. The aim of our study was to investigate the risk factors for manipulation after primary TKA and further investigate the risk of subsequent revision TKA.

## Methods

### Study design

The data used in this study was initially collected from the national registers for the PERFECT (PERFormance, Effectiveness, and Cost of Treatment episodes) project. Data included information from the Finnish Arthroplasty Register (FAR) and the Care Register of Health Care (CRHC) from the Finnish Institute for Health and Welfare, drug reimbursement data from the Social Insurance Institution, and the Causes of Death data from Statistics Finland [19]. The FAR alone captures about 95% of primary TKAs and 85% of revision TKAs (https://www.thl.fi/far), but by combining data from the FAR and CRHC virtually all primary and revision TKAs can be identified [20,21].

The study is reported according to STROBE guidelines.

### Data

We included first TKA performed between January 1999 and December 2020 in Finland. We excluded patients under 40 years of age at the time of TKA. The data did not enable us to identify a specific indication for TKA for all patients. However, at a population level, about 95% of patients had osteoarthritis as an indication for TKA. As the primary objective of this study was to analyze the risk of manipulation in patients with primary osteoarthritis, an age threshold of over 40 years was applied, because primary osteoarthritis is rare in individuals younger than 40 years.

Manipulation was defined as mobilization of the knee joint under either general or spinal anesthesia and was identified from the registers using the NCSP (Nordic Classification of Surgical Procedures) code NGT60. It is known that manipulations performed before 1 week or after 1 year from the day of TKA are rare [2]. Therefore, we included primary manipulations that were performed between 1 week and 1 year from the day of TKA.

In the PERFECT project, several comorbidities had also been identified from the registers for the purpose of risk adjustment [19]. These included hypertension, coronary artery disease, atrial fibrillation, heart failure, diabetes mellitus, hypercholesterolemia, depression, psychosis, Parkinson’s disease, dementia, alcohol or drug dependency, cancer, and chronic obstructive pulmonary disease or asthma. Data on clinical measurements, patient-reported outcome measures (PROM), BMI, or range of motion was not available in the database and was therefore not considered in the present study.

Revision TKAs were defined as reoperations of a prosthetic knee where prosthetic parts are inserted, replaced, added, or removed (including arthrodesis and amputation), as defined in the FAR.

### Statistics

The cumulative incidence of manipulation and revision TKA was estimated using the Aalen–Johansen estimator, which appropriately accounts for competing events. Follow-up for the cumulative incidence of manipulation started at the date of first primary TKA (index day). Death and revision TKA were considered competing risks, and follow-up time for this model was restricted to 1 year. For 163 (4.1%) patients who had manipulation within 1 year from first TKA, contralateral TKA was performed before manipulation. As the laterality of manipulation was unknown, it could not be determined whether manipulation was performed on the index knee or the contralateral knee if the contralateral TKA preceded manipulation. Therefore, these patients were censored at the time of contralateral TKA. Follow-up for cumulative incidence of revision TKA started at the date of manipulation (index day). Death before revision was considered as a competing risk, and patients were censored at contralateral TKA or the end of the study period.

To assess the relative risk of revision TKA, we used a cause-specific Cox proportional hazards model, with manipulation included as a time-dependent exposure. Patients entered the manipulation-exposed risk set at the time of manipulation. Both univariate and multivariable models were fitted, adjusting for all relevant covariates, to estimate hazard ratios (HRs) and 95% confidence intervals (CIs). Separate Cox models were constructed for the risk of manipulation and the risk of revision TKA. As the study spans decades, all Cox models are stratified by year of TKA. Statistical analyses were performed with the R software package v. 4.2.2 (R Foundation for Statistical Computing, Vienna, Austria).

### Ethics, registration, data sharing plan, funding, and disclosures

Permission to use the register data was obtained from the Finnish Institute for Health and Welfare (Dnro THL/7019/6.02.00/2020). The PERFECT project had previously been approved by the ethics committee of the same institution (THL 1406/6.02.00/2009). This study did not receive any form of grants or funding. Due to privacy regulations, the original data cannot be shared. Anonymized summary tables can be shared on reasonable request. The authors declare no conflict of interest. Complete disclosure of interest forms according to ICMJE are available on the article page, doi: 10.2340/17453674.2026.46072

## Results

**Figure 1:**
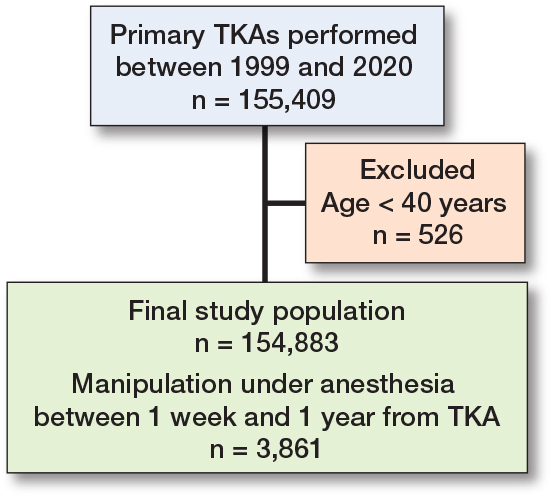
Flowchart of the study population. TKA = total knee arthroplasty.

During the study period, 154,883 patients undergoing primary TKA were identified for the final sample, of whom 3,861 underwent primary manipulation within 1 week to 1 year after TKA. The flowchart of the study population is shown in Figure 1. At the time of primary TKA, the mean age was 62.8 years (SD 9.2) in patients undergoing manipulation within 1 year of TKA and 68.3 years (SD 9.4) in patients without manipulation within 1 year of TKA.

### Risk factors for manipulation under anesthesia

**Figure 2:**
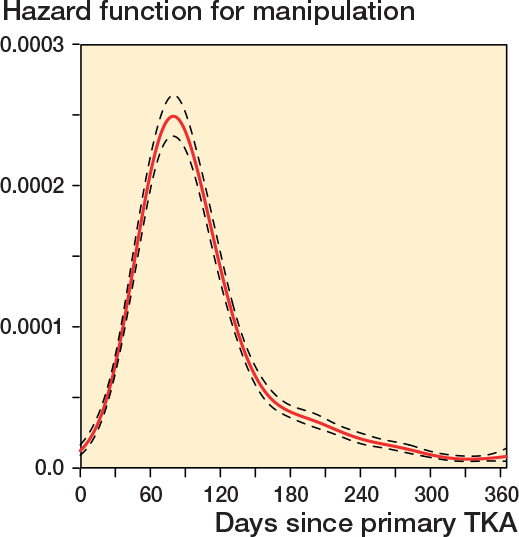
Hazard function for manipulation-under-anesthesia with 95% confidence interval between 1 week and 1 year after total knee arthroplasty (TKA) (probability that manipulation took place on a certain day but had not previously occurred). Median time to manipulation was 92 days.

The 1-year cumulative incidence of manipulation was 2.49% (CI 2.42–2.57). The mean time interval between primary TKA and the first manipulation performed was 15.0 weeks (SD 8.9). The hazard function for manipulation occurrence, which describes the probability of manipulation at a certain moment on the condition that it has not happened so far, illustrates that probability of manipulation rises from day 1 to about 3 months after primary TKA, then quickly decreases until 6 months, continues a slower decrease until about 10 months, and then stabilizes to a very rare level; based on the area under the curve, 88% of manipulations were performed within 6 months of primary TKA (Figure 2).

**Figure 3:**
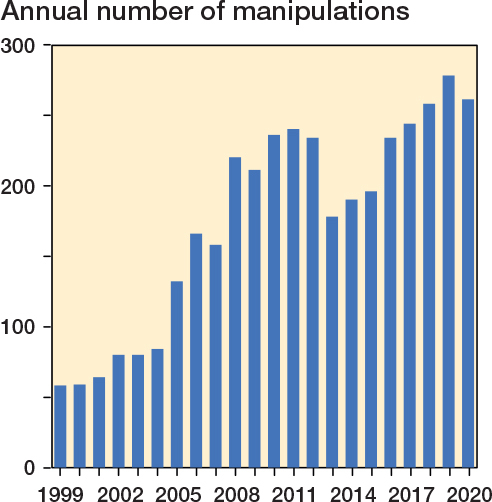
Annual distribution of manipulation under anesthesia in 3,861 primary manipulation procedures performed between 1 week and 1 year from primary total knee arthroplasty.

The annual distribution of manipulation under anesthesia in the 3,861 primary manipulations performed between 1 week and 1 year from primary total knee arthroplasty showed a steady increase from 55 to 250 during the study period 1999 to 2020 (Figure 3).

Patients requiring manipulation were more often female than male (n = 2,755, CIF 2.71% [CI 2.61–2.81] vs n = 1,106, CIF 2.07% [CI 1.96–2.20], respectively) and relatively younger. Female sex was associated with an increased risk of manipulation in both the univariate (HR 1.30, CI 1.21–1.40) and the multivariable analysis (HR 1.53, CI 1.42–1.64). Increasing age was associated with a decreased risk of manipulation in both the univariate (HR 0.94 per year, CI 0.94–0.95) and the multivariable analysis (HR 0.94 per year, CI 0.94–0.94). Diabetes mellitus (multivariable HR 1.19, CI 1.08–1.31), coronary artery disease (multivariable HR 1.25, CI 1.12–1.39), and hypercholesterolemia (multivariable HR 1.16, CI 1.06–1.28) were associated with an increased risk of manipulation in the multivariable analysis but not in the univariate analysis (Table 1). Hypertension (multivariable HR 0.78, CI 0.73–0.84), depression (multivariable HR 0.78, CI 0.70 to 0.87), psychosis (multivariable HR 0.69, CI 0.56–0.85), and alcohol or drug dependency (multivariable HR 0.59, CI 0.45–0.78) were associated with a decreased risk of manipulation in the univariate and the multivariable analyses (Table 1). Atrial fibrillation, heart failure, Parkinson’s disease, dementia, and cancer were associated with a decreased risk of manipulation in the univariate analysis but not in the multivariable analysis (Table 1).

**Table 1:** Risk of manipulation according to multiple factors using the Cox proportional hazard model

Factor	n (%)	HR (CI) univariate	HR (CI) multivariable
Age, mean (SD) = 68.2 (9.4)	0.94 (0.94–0.95)	0.94 (0.94–0.94)	
Sex			
Male	53,337 (34)		
Female	101,546 (66)	1.30 (1.21–1.40)	1.53 (1.42–1.64)
Hypertension			
No	76,960 (50)		
Yes	77,923 (50)	0.66 (0.61–0.70)	0.78 (0.73–0.84)
Coronary artery disease			
No	132,423 (85)		
Yes	22,460 (15)	0.75 (0.68–0.83)	1.25 (1.12–1.39)
Atrial fibrillation			
No	141,033 (91)		
Yes	13,850 (8.9)	0.75 (0.66–0.85)	1.08 (0.95–1.23)
Heart failure			
No	149,287 (96)		
Yes	5,596 (3.6)	0.61 (0.49–0.75)	1.05 (0.84–1.31)
Diabetes mellitus			
No	132,704 (86)		
Yes	22,179 (14)	1.02 (0.93–1.12)	1.19 (1.08–1.31)
Hypercholesterolemia			
No	130,913 (85)		
Yes	23,970 (15)	1.00 (0.92–1.09)	1.16 (1.06–1.28)
Depression			
No	136,608 (88)		
Yes	18,275 (12)	0.88 (0.79–0.97)	0.78 (0.70–0.87)
Psychosis			
No	149,133 (96)		
Yes	5,750 (3.7)	0.67 (0.55–0.82)	0.69 (0.56–0.85)
Parkinson’s disease			
No	151,969 (98)		
Yes	2,914 (1.9)	0.69 (0.52–0.91)	0.88 (0.67–1.17)
Dementia			
No	153,284 (99)		
Yes	1,599 (1.0)	0.45 (0.28–0.71)	0.82 (0.51–1.30)
Alcohol or drug dependency			
No	151,668 (98)		
Yes	3,215 (2.1)	0.68 (0.52–0.88)	0.59 (0.45–0.78)
Cancer			
No	136,899 (88)		
Yes	17,984 (12)	0.76 (0.68–0.85)	0.98 (0.88–1.10)
COPD or asthma			
No	130,100 (84)		
Yes	2,4783 (16)	1.00 (0.92–1.09)	0.97 (0.89–1.06)

Univariate and multivariable Cox proportional hazard model for the associations between the factors and the risk of manipulation. The analyses were stratified by the year of the TKA.

HR = hazard ratio; CI = 95% confidence interval; COPD = chronic obstructive pulmonary disease.

### Risk of revision TKA

**Figure 4:**
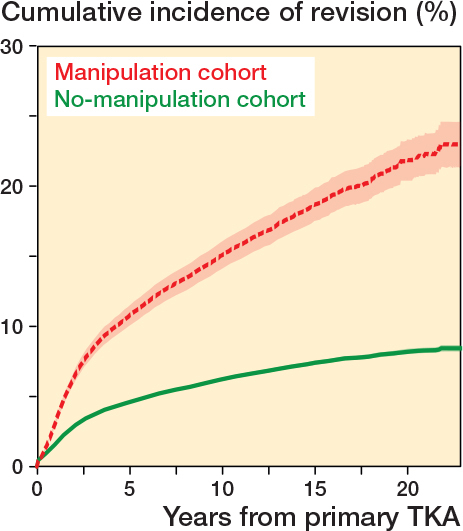
Cumulative incidence of revision total knee arthroplasty (TKA) with manipulation as a time-dependent covariate. Manipulation increased the risk of revision TKA significantly (HR 2.26, CI 2.05–2.50).

**Figure 5:**
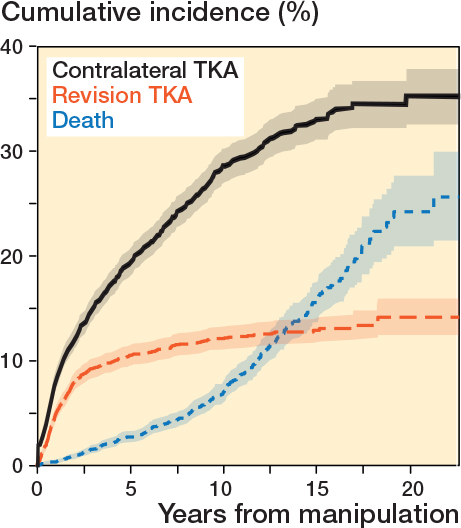
Cumulative incidence of contralateral TKA, death, and revision TKA after manipulation. Follow-up started from the day of manipulation. Revision TKA was the event of interest, and death before revision was considered as a competing risk. Patients were censored at the end of follow-up. TKA = total knee arthroplasty.

Patients requiring manipulation within 1 year of primary TKA had a significantly increased risk of revision TKA, calculated from the date of the manipulation onwards (HR 2.26, CI 2.05–2.50). The 10-year cumulative risk of revision TKA after manipulation was 15.0% (CI 14.1–16.0) (Figure 4). Increasing age was associated with a decreased risk of revision TKA after manipulation. Coronary artery disease and Parkinson’s disease were associated with an increased risk of revision TKA after manipulation. However, the number of patients with Parkinson’s disease was relatively small, but 50 patients underwent manipulation, of whom 15 subsequently required revision TKA. The patient’s sex was not associated with the risk of revision TKA after manipulation. 736 patients (19%) underwent manipulation within 8 weeks of TKA, 925 (24%) between 8 and 12 weeks, and 2,200 (57%) after 12 weeks. Manipulation performed between 8 and 12 weeks was statistically significantly associated with a lower risk of revision TKA compared with manipulation before 8 weeks (HR 0.72, CI 0.54–0.96). Additionally, contralateral TKA was relatively unlikely to be performed for those patients who required manipulation within 1 year of TKA (multivariable HR 0.77, CI 0.72–0.82). The results of the Cox proportional hazard model for the associations between the factors and the risk of revision TKA in patients requiring manipulation are presented in Table 2. The cumulative incidence of death, revision TKA, and contralateral TKA after manipulation are shown in Figure 5.

**Table 2:** Risk of revision TKA after manipulation according to multiple factors using the Cox proportional hazard model

Factor	n (%)	HR (CI) univariate	HR (CI) multivariable
Age, mean (SD) = 62.8 (9.2)	0.99 (0.98–1.00)	0.98 (0.97–0.99) **^a^**	
Sex			
Male	1,106 (29)		
Female	2,755 (71)	1.05 (0.85–1.30)	1.05 (0.85–1.31)
Timing of manipulation			
< 8 weeks	736 (19)		
8–12 weeks	925 (24)	0.67 (0.50–0.88)	0.72 (0.54–0.96)
> 12 weeks	2,200 (57)	0.78 (0.62–0.98)	0.84 (0.66–1.06)
Hypertension			
No	2,315 (60)		
Yes	1,546 (40)	1.04 (0.86–1.26)	1.01 (0.81–1.25)
Coronary artery disease			
No	3,424 (89)		
Yes	437 (11)	1.26 (0.96–1.66)	1.41 (1.03–1.92)
Atrial fibrillation			
No	3,599 (93)		
Yes	262 (6.8)	0.97 (0.65–1.45)	1.09 (0.71–1.66)
Heart failure			
No	3,776 (98)		
Yes	85 (2.2)	0.64 (0.29–1.44)	0.59 (0.26–1.35)
Diabetes mellitus			
No	3,301 (85)		
Yes	560 (15)	1.17 (0.90–1.52)	1.24 (0.94–1.64)
Hypercholesterolemia			
No	3,258 (84)		
Yes	603 (16)	1.00 (0.78–1.29)	1.01 (0.76–1.34)
Depression			
No	3,455 (89)		
Yes	406 (11)	0.96 (0.71–1.31)	0.90 (0.65–1.25)
Psychosis			
No	3,764 (97)		
Yes	97 (2.5)	1.15 (0.65–2.04)	1.22 (0.66–2.23)
Parkinson’s disease			
No	3,811 (99)		
Yes	50 (1.3)	1.92 (1.06–3.50)	1.86 (1.01–3.42)
Dementia			
No	3,843 (99)		
Yes	18 (0.5)	1.14 (0.29–4.59)	1.13 (0.28–4.61)
Alcohol or drug dependency			
No	3,807 (99)		
Yes	54 (1.4)	0.34 (0.09–1.37)	0.33 (0.08–1.36)
Cancer			
No	3,510 (91)		
Yes	351 (9.1)	1.15 (0.83–1.58)	1.28 (0.92–1.78)
COPD or asthma			
No	3,244 (84)		
Yes	617 (16)	1.14 (0.89–1.46)	1.13 (0.88–1.45)

a per year.

Univariate and multivariable Cox proportional hazard model for the associations between the factors and the risk of revision TKA in patients requiring manipulation. The analyses were stratified by the year of the TKA.

For abbreviations, see Table 1.

## Discussion

We aimed to investigate the risk factors for manipulation after primary TKA and for the subsequent revision TKA in patients requiring manipulation using national healthcare registers. We found that younger age, female sex, coronary artery disease, diabetes mellitus, and hypercholesterolemia increased the risk of manipulation in the multivariable analysis. Increased risk of manipulation in younger TKA patients has been reported previously [3-6,8,9,11]. Younger patients are more likely to have higher expectations for knee function and may therefore have undergone manipulation with a better range of motion than older patients. Notably, the inclusion criterion was age over 40. Additionally, females had an increased risk of manipulation. Several previous studies have noted that manipulations were performed more often for female patients than male patients, but the underlying reason is unknown [5,8,9,12]. We found that diabetes mellitus increased the risk of manipulation in the multivariable analysis but not in the univariate analysis. According to previous studies, the role of diabetes mellitus for stiffness is unclear, with evidence of increased risk of manipulation [11,13], no association with risk of manipulation [3,4,8], and also decreased risk of manipulation [9]. Conflicting factors other than those factors included in this study may have affected the results. For instance, there is a correlation between diabetes mellitus and obesity [22] and previous studies have found that obesity increases the risk of manipulation [8,12]. Thus, the effect of diabetes mellitus on the risk of manipulation is difficult to determine. In this study, we did not have access to BMI data.

Coronary artery disease was associated with an increased risk of manipulation in the multivariable analysis, which may be explained by less compliance with postoperative physical therapy. Additionally, smoking is a known risk factor for coronary artery disease and stiffness after TKA [3,4,8,9,11]. Consequently, this might partly explain the association between coronary artery disease and increased risk of revision TKA. In addition, many coronary-artery-disease patients use anticoagulant or antithrombotic medications, which increases the risk of postoperative hematomas and, consequently, may lead to delayed mobilization, joint stiffness, and arthrofibrosis [23]. Werner et al. found an association between cardiovascular diseases (hyperlipidemia, hypertension, congestive heart failure, and coronary artery disease) and a decreased risk of manipulation [9]. In this study, hypertension was associated with a decreased risk of manipulation, supporting the results of Werner et al. [9]. We found that depression, psychosis, Parkinson’s disease, dementia, and alcohol or drug dependency were associated with a decreased risk of manipulation. It is possible that patients with the aforementioned conditions might have lower expectations regarding knee range of motion, leading to a mutual decision between the patient and the surgeon not to conduct manipulation. It was also noted that patients with Parkinson’s disease were at a higher risk of complications after TKA [24], and therefore conservative treatment may often have been chosen instead of manipulation. Thus, such conditions may not be considered as protective factors for knee stiffness. Pfefferle et al. found no difference in the frequency of manipulation regardless of whether a patient had depression and/or opioid dependence/abuse [8]. No association was found between lung diseases (chronic obstructive pulmonary disease or asthma) and the risk of manipulation, which is in line with the previous knowledge [9]. The indication for TKA was not available in the dataset; however, given that the inclusion criterion was age over 40 years, most TKAs were likely performed for primary osteoarthritis. In addition, previous studies found that low preoperative range of motion, smoking, high BMI, and previous knee surgery increased the risk of manipulation [3-5,8,9,11,12]. We did not have data on the factors mentioned above. We noticed that the 1-year cumulative incidence of manipulation was 2.49%. This finding is consistent with the previous studies [2-9].

Our study showed that in patients requiring manipulation within 1 year after primary TKA, the risk of revision TKA was significantly increased compared with patients who did not require manipulation (HR 2.26). This finding is consistent with the previous studies. Thorsteinsson et al. found in a Swedish national register study that patients requiring manipulation had approximately double the 10-year cumulative revision rate [5]. Furthermore, Werner et al discovered that patients who had manipulation within 6 months of TKA were at a 2.43-fold risk of revision TKA compared with those who did not in the following 7 years [9]. Parkulo et al. found that the risk of revision TKA was significantly higher in patients who had manipulation compared with the patients who did not at 1-, 2-, and 5-year time points with an HR of 3.81, 3.90, and 3.22, respectively [25]. Consequently, revisions were performed more often for patients who had manipulation after primary TKA, but this finding is only evidence of association, not causality. There are some rare complications of manipulation, for example, femoral or tibial fractures, patellar tendon ruptures, incision ruptures, and hemarthrosis [26,27]. In some cases, complications of manipulation can lead to revision TKA, but because they are relatively rare, they do not merely explain the significantly increased risk of revision TKA in manipulation patients.

Nevertheless, manipulation improves the range of motion in the majority of patients but not all achieve a satisfactory range of motion after manipulation [2,6,7]. In these cases, the cause of stiffness is more likely other than arthrofibrosis, such as malposition and/or oversizing of TKA components or inadequate osteophyte removal [14]. These patients might need revision TKA after failed manipulation. Thus, no firm conclusions could be deduced between the manipulation procedure itself and revision TKA. Unfortunately, we did not have comprehensive data considering the indications for revisions in this register-based study. In the previous studies, the indication for revision TKA after manipulation was more often mechanical and less often infection-related compared with the general TKA population [5,9,17]. Further studies are needed to demonstrate whether there is any causality between the manipulation procedure itself and the need for revision TKA.

We found that manipulation between 8 and 12 weeks from TKA was associated with decreased risk of revision TKA compared with early manipulation. On the other hand, manipulation before 8 weeks from TKA increased the risk of revision TKA. The findings are in contradiction with Thorsteinsson et al., who reported no difference in whether manipulation was performed before or after 8 weeks from TKA [5]. However, Werner et al. showed a potential association, with reported revision rates of 3.8% for manipulation within 8 weeks and 5.3% for manipulation between 8 weeks and 3 months after TKA (9). We also found no patient sex-based differences in revision rates, supporting Thorsteinsson et al. [5], though Brigati et al. reported male sex as a risk factor for revision after manipulation [17]. We noticed that younger patients were at increased risk of revision TKA after manipulation, which is in line with Werner et al. [9], but contrasts with Thorsteinsson et al. [5]. This may reflect higher functional expectations among younger patients. Additionally, patients with Parkinson’s disease had an increased risk of revision TKA after manipulation, consistent with Rondon et al., who found elevated complication and revision rates in Parkinson’s disease patients undergoing TKA [24]. However, the small number of Parkinson’s disease patients in this study may predispose the analysis to sparse-data bias. In this study, we found that contralateral TKA was relatively less often performed for those patients who required manipulation during the study period. This is an expected outcome, as stiffness prolongs the rehabilitation process and makes patients less satisfied with the outcome of TKA [28].

### Limitations

The database we used did not include information on some potential risk factors for manipulation such as BMI, preoperative range of motion, and smoking. Additionally, we did not analyze the use of a tourniquet during primary TKA. Moreover, the case-level indication for TKA (at the population level about 95% of cases had osteoarthritis as an indication) was missing, and the laterality of manipulation remained unclear in 163 (4.1%) cases. Additionally, the register data did not include the specific indications for revisions after manipulation.

### Conclusion

We showed that females, younger patients, and patients with diabetes mellitus, coronary artery disease, and hypercholesterolemia had an increased risk of manipulation after TKA. Patients who had manipulation within 1 year of TKA were at elevated risk of ending up with revision TKA, with younger age, Parkinson’s disease, and coronary artery disease associated with the increased risk.

*In perspective*, patients with risk factors for stiffness should be monitored, and if necessary, rehabilitation should be intensified to prevent the development of stiffness.

## Figures and Tables

**Figure:**
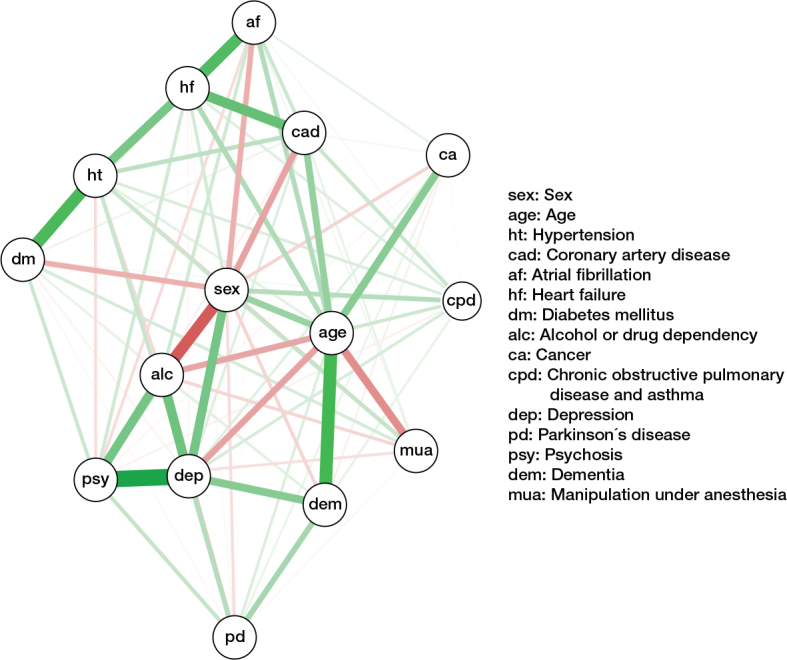
Partial correlations of assessed factors.

## Notes

All authors contributed to the planning of the study. RS collected and analyzed the data. All authors interpreted the results. JS (Sala) wrote the article. RS, JS (Sirola), AJ, and HK assisted in the writing process.

Handling co-editors: Per Henrik Randsborg and Robin Christensen

*Acta* thanks Michael Axenhus and Annette W-Dahl for help with peer review of this manuscript.
